# 

**Published:** 1908-06-15

**Authors:** 


### ANNOUNCEMENTS.

NATIONAL DENTAL ASSOCIATION.—The 12th annual meeting of the association will be held in Boston on July 28th to 31st, inclusive, and promises to be one of the most important in the history of the society. It is 28 years since our National Society has held a meeting in New England and it is confidently predicted that the attendance and interest will surpass that of any previous session.

Hotel Somerset, on Commonwealth ave., has been selected as headquarters for the association and where all meetings will be held, including those of sections. The clinics will be held in Tuft's College Dental School, on Huntington ave., on the forenoons of Wednesday and Thursday.

Rates at Hotel Somerset:

Two in room with bath, $4.00 per day. One in room with bath, $3.00 per day. Two in room without bath, $3.50 per day. One in room without bath, $2.50 per day.

For reservations, etc. apply to chairman of the local committee of arrangements, Dr. W. E. Boardman, 419 Boylston Street.

Membership in the association is limited to delegates from state societies, but a most cordial invitation is extended to all reputable dentists to attend the meeting.

DR. WILLIAM CARR, *President*,
35 W. 46th st., N. Y.
DR. CHAS. S. BUTLER, *Secretary*.
Buffalo, N. Y.

---

INTERSTATE DENTAL FRATERNITY.—The Board of Governors of the Interstate Dental Fraternity will convene for the annual business meeting of the Order in Boston at the Brunswick, Boylston st., July 24th. The annual banquet will occur during the week and due notice thereof will be sent to the members as soon as arrangements can be made and the exact date fixed. It is hoped that the Fraternity will meet in large numbers on this occasion.

DR. R. M. SANGER, *Nat. Secty*.

East Orange, N. J.

---

MONTANA STATE BOARD OF DENTAL EXAMINERS.—The annual meeting of the Montana State Board of Dental Examiners will be held in Helena, commencing the second Monday in July, 1908, and continuing three days. All applications, and fee of twenty-five dollars, should be filed at least ten days prior to the meeting. Application blanks, and the dental laws of Montana, which every applicant is expected to read before the examination, will be furnished upon application to the secretary. Vulcanizers, and dental engines, without handpieces, will be furnished.

D. J. WAIT, *Secretary*,
103 Broadway.

---

PENNSYLVANIA STATE DENTAL SOCIETY.—The fortieth annual meeting of the Pennsylvania State Dental Society will be held at the Bellevue Stratford Hotel, Philadelphia, Pa., on June 30, July 1 and 2, 1908, and it is the intention of every officer and committeeman of this society to make this anniversary the best of its kind in the dental history of the state.

---

CADILLAC (MICH.) DENTAL SOCIETY elected the following officers: Dr. F. R. Fletcher, president; Dr. Howard F. Kneeland, vice-president; Dr. A. W. Knight, secretary and treasurer. Professional advancement and co-operation are the objects. It is the expectation to affiliate with the state dental society.

---

SOUTH DAKOTA STATE BOARD OF DENTAL EXAMINERS.—The next meeting of the South Dakota State Board of Dental Examiners will begin Monday, July 20, 1908, beginning promptly at 9 o'clock A. M. and continuing three days, at Lead, S. D. All persons desiring to take this examination must make application to the secretary, and send fee of $10.00 at least one week prior to the above date. No candidates will be received for examination who do not make application as above specified. Applicants are required to bring dental engine, filling materials, articulators, teeth, and all appliances and materials necessary to do crown and bridge work.

G. W. COLLINS, *Secretary.*

---

EIGHTH DISTRICT SOCIETY OF MICHIGAN elected the following officers: President, Dr. W. R. Purmort, Saginaw; vice-president, Dr. H. B. Hulbert, Bay City; secretary, Dr. W. E. Moore, Saginaw; treasurer, Dr. C. J. Hand, Bay City.

---

VIRGINIA STATE DENTAL ASSOCIATION.—The meeting place of the Virginia State Dental Association has been changed to Richmond, Va., Murphy's Hotel Annex, July 14, 15, 16, 1908.

W. H. PEARSON,
*Corresponding Secretary.*

